# Lactose-Induced Chronic Diarrhea Results From Abnormal Luminal Microbial Fermentation and Disorder of Ion Transport in the Colon

**DOI:** 10.3389/fphys.2020.00877

**Published:** 2020-07-29

**Authors:** Hong Xue, Min Zhang, Jinxin Ma, Ting Chen, Fengyun Wang, Xudong Tang

**Affiliations:** Digestive Laboratory of Traditional Chinese Medicine Research Institute of Spleen and Stomach Diseases, Xiyuan Hospital, China Academy of Chinese Medical Sciences, Beijing, China

**Keywords:** lactose, diarrhea, microbiota, SCFA, Na^+^

## Abstract

Diarrhea is one of the major abdominal symptoms in lactose-intolerant subjects. The changes in the large intestinal luminal environment and disorder of the epithelial ion transport in lactose-induced diarrhea remain unclear. The present study aimed to investigate the effect of an incremental high-lactose diet (IHLD, 30%/40%/50%) on luminal microbiota, microbiota-derived metabolite concentrations and colonic ion transport. Gut microbiota were analyzed by 16S rRNA amplicon sequencing and the concentration of SCFAs by gas chromatography, galactose, lactose and lactic acid through assay kit; Ussing chamber was performed to detect basal and stimulated ion transport; The expression and location of SCFA transporters, the Na-H exchanger 3(NHE3), cystic fibrosis transporter regulater (CFTR) and NKCC1 in the colon mucosa were analyzed by western and immunostaining. The concentrations of lactose, galactose and lactic acid of the cecal content were markedly increased (*P* < 0.01) and SCFA concentration was significantly decreased (*P* < 0.01). This was associated with depletion of the Lachnospiraceae NK4A136 group and Ruminococcaceae UCG-005 and increased relative abundance of Lactobacillus, escherichia-shigella and megamonas in the cecal microbiota. The expression of monocarboxylate transporter 1 was decreased in the colonic mucosa of the IHLD group. Low NHE3 expression and phosphorylation levels, and decreases in delta basal short circuit current after apical Na^+^ removal in the colonic mucosa of the IHLD group contributed to Na^+^ accumulation in the lumen and decrease stimulated Cl^–^ secretion with low CFTR and NKCC1 expression would compensate for water and electrolyte loss during the diarrhea process. These results indicated that the persistence of the diarrhea state was maintained by abnormal colonic microbiota fermentation leading to high concentrations of lactose, galactose and lactic acid and low SCFAs in the lumen, and decreased Na^+^ absorption with the low NHE3 expression and phosphorylation levels.

## Introduction

Diarrhea is a highly prevalent and bothersome disorder, particularly in children and the elderly. According to the WHO, it is estimated that 577,000 children aged <5 years and 502,000 adults aged >70 years will die from diarrheal diseases worldwide (Thapar and Sanderson, 2004; Bryce et al., 2005; Wardlaw et al., 2010). Long-standing diarrhea causes malnutrition, which in turn leads to more difficulty in treating. Approximately 70% of the world’s population has hypolactasia after weaning, resulting in lactose malabsorption or lactose intolerance, defined as the presence of gastrointestinal symptoms such as bloating, rumbling, abdominal pain, nausea and diarrhea (Swagerty et al., 2002; Bayless et al., 2017; Fassio et al., 2018).

As we know, lactose intolerance-induced diarrhea belongs to osmotic diarrhea, thereby resulting in colonic water and electrolyte accumulation (Hammer and Hammer, 2012). It is believed that the osmotic load is not only caused by undigested lactose but that the colonic fermentation capacity plays a role in lactose intolerance (He et al., 2006). Lactose is fermented by the colonic resident microbiota into a series of intermediate and end-product metabolites, including lactate, formate, succinate and short-chain fatty acids (SCFAs), including acetate, propionate and butyrate (He et al., 2006; Windey et al., 2015). Some reports have indicated that the osmolality of lactose-induced diarrhea is closely linked to the lactose content and the production of SCFAs in the colon (Binder, 2010; Alexandre et al., 2013). SCFAs are the main anions of the colon, which have been demonstrated to be novel regulators of colonic ion transport (Yajima et al., 2011; Kaji et al., 2016). Therefore, we speculated that the mechanism of lactose-induced diarrhea involved a change in SCFAs or lactate leading to a disturbance in colonic epithelial ion transport resulting in diarrhea. In the process of the transport of SCFAs, monocarboxylate (MCT1) and sodium MCT1 (sMCT1) transporters play an important role in mediating SCFA influx into the blood from the lumen (Ritzhaupt et al., 1998; Ganapathy et al., 2005; Sivaprakasam et al., 2017).

It is commonly recognized that diarrhea results from excessive secretion and/or impaired absorption of fluid and electrolytes across the intestinal epithelium. Excessive fluid secretion is caused by active chloride secretion into the intestinal lumen, which drives secondary movement of sodium and water (Binder et al., 1991; Hoque et al., 2012). Chloride secretion involves the activation of chloride channel(s), such as CFTR, on the apical plasma membrane, and it is transported into the cell at the basolateral membrane by a Na/K/Cl symporter (NKCC1, also known as SLC12A2) (Kunzelmann and Mall, 2002). Na^+^ absorption includes electrogenic Na^+^ absorption mediated by epithelial Na^+^ channels (ENaC), whereas electroneutral Na^+^ absorption is mediated by the Na-H exchanger (NHE) and Cl^–^-HCO${}_{3}^{-}$ exchanger (an anion exchanger; AE) (Binder et al., 1987). NHE3 mediates both HCO_3_-dependent and butyrate-dependent Na^+^ absorption in the normal gut, which is regulated by butyrate (Subramanya et al., 2007), inducing NHE3 expression and increasing Na^+^ absorption in the rat colon (Musch et al., 2001; Vidyasagar and Ramakrishna, 2002).

Although some studies demonstrated that undigested lactose and SCFAs induce diarrhea secondary to osmotically induced accumulation of ions and water in the colon (Christopher and Bayless, 1971; Hammer et al., 1989), the change in the large intestinal luminal environment and disorder of the epithelial ion transport mechanism of lactose-induced chronic diarrhea remained unclear. Therefore, the objective of the study was to investigate the effect of an incremental high-lactose diet (IHLD, 30%/40%/50%) on luminal microbiota, microbiota-derived metabolite concentrations and colonic ion transport. The present study provides new evidence for the physiopathological mechanism of lactose intolerance-induced high osmosis diarrhea and new ideas for therapy through modulating the gut microbiota and Na^+^ absorption.

## Materials and Methods

### Animals and Diarrhea Induction Using an Incremental High Lactose Diet

**FIGURE 1:**
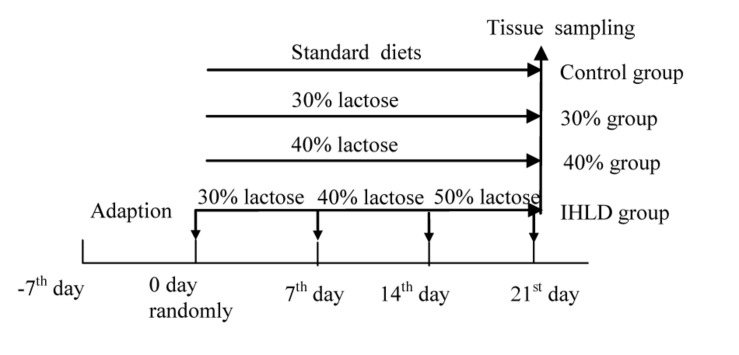
Plan of experimental animal grouping showing the days for different diets feeding.

The experimental procedures followed the guidelines and practices of the Animal Care Ethics Committee of Xiyuan Hospital (Permission code: 2019XLC004-2). The procedures were conducted in accordance with the Beijing Administration Office Committee of Laboratory Animals. Male Wistar rats (350–400 g) were housed under an artificial 12 h light-12 dark cycle (lights on at 08:00 h). Forty rats were fed a standard chow diet *ad libitum* and had free access to water for 4 days prior to the induction of diarrhea. Then, all rats were randomly divided into 4 groups of 10 animals each. The control group was fed a standard chow diet; the 30% lactose group was fed 30% lactose for 3 weeks; the 40% lactose group was fed 40% lactose for 3 weeks; and the IHLD group was fed 30–40–50% IHLD, which consisted of 30% lactose for the first week, 40% lactose for the second week and 50% lactose for the third week (Figure 1). An IHLD containing 30, 40, and 50% lactose in place of starch was fed to rats (Keao Xieli Feed Co Ltd., Beijing). Rats were monitored for changes in the consistency of pellets and stool mass and fecal fluid. Rats were considered diarrheic if they produced watery, soft, yellowish stools compared to normal, pliable, soft, well-formed pellets as previously described by other researchers (Boakye et al., 2012).

### DNA Extraction and PCR Amplification

Cecal microbiota DNA was extracted using the Omega Stool DNA Kit (Qiagen, United States) and applied to amplification of the V3-V4 region of 16S rDNA with the gene-specific primers ACTCCTACGGGAGGCAGCAG and GGACTACHVGGGTWTCTAAT.PCR reactions were performed on a Mastercycler Gradient (Eppendorf, Germany) using 25 μl reaction volumes containing 12.5 μl of 2× Taq Plus Master Mix, 1 μl of forward primer (5 μM), 1 μl of reverse primer (5 μM), 3 μL of BSA (2 ng/μL), 5 μl of DNA (total template quantity is 30 ng), and 2.5 μl of ddH_2_O. PCR amplification was as follows: initiation at 94°C for 5 min, followed by 28 cycles at 94°C for 30 s, 55°C for 30 s, and 72°C for 60 s, with a final extension of 72°C for 7 min. Three PCR products per sample were pooled to mitigate reaction-level PCR biases. The PCR products were purified using a QIAquick Gel Extraction Kit (QIAGEN, Germany), quantified using Real Time PCR, and sequenced at Allwegene Company, Beijing.

### High-Throughput Sequencing

Deep sequencing was performed on the MiSeq platform at Allwegene Company (Beijing). After the run, image analysis, base calling and error estimation were performed using Illumina Analysis Pipeline Version 2.6.

### Data Analyses

The raw data were first screened, and sequences were removed from consideration if they were shorter than 200 bp, had a low quality score (≤20), contained ambiguous bases or did not exactly match primer sequences and barcode tags. Qualified reads were separated using the sample-specific barcode sequences and trimmed with Illumina Analysis Pipeline Version 2.6. Then, the dataset was analyzed using QIIME. The sequences were clustered into operational taxonomic units (OTUs) at a similarity level of 97% (Edgar, 2013) to generate rarefaction curves and to calculate the richness and diversity indices. The Ribosomal Database Project (RDP) Classifier tool was used to classify all sequences into different taxonomic groups (Cole et al., 2009).

To examine the similarity between different samples, clustering analyses and PCA were used based on the OTU information from each sample using R (Wang et al., 2012). The evolution distances between microbial communities from each sample were calculated using the tayc coefficient and represented as an Unweighted Pair Group Method with Arithmetic Mean (UPGMA) clustering tree describing the dissimilarity (1-similarity) between multiple samples (Jiang et al., 2013). A Newick-formatted tree file was generated through this analysis. To compare the membership and structure of communities in different samples, heat maps were generated with the top 20 OTUs using Mothur (Jami et al., 2013).

### SCFA, Lactose, Galactose and Lactic Acid Quantity

The cecal contents were simply sampled in sterile tubes. Samples were kept frozen at −80°C until analysis. The samples were removed from the freezer, and 1,200 μL of water was added to each thawed sample. The samples were vortexed for 1 min until the material was homogenized. The SCFA content was determined by gas chromatography. Chromatographic analysis was carried out using an Agilent GC system with a flame ionization detector (FID) (Shimadzu Corp, Kyoto, Japan). Fused silica capillary columns 30 m × 0.25 mm coated with 0.25 μm film thickness were used. Nitrogen was used as the carrier gas. The oven temperature was 250°C, and the FID and injection port were set to 300 and 250°C, respectively. The injected sample volume was 1 μL, and the run time for each analysis was 32 min. The chromatograms and data integration were carried out using a Shimadzu C-R5A Chromatopac. The method was performed according to reference (David et al., 2014).

Lactose, galactose and lactic acid were detected by corresponding assay kits (ELAC-100, EGAL-100 and ECLC-100, respectively, Bioassay Systems, Hayward, CA, United States) according to the instructions. An aliquot of 50 mg of cecal and proximal colon content samples was dissolved in deionized water, vortexed for 20 s and centrifuged at 12,000 × *g* for 10 min at 4°C. The supernatant was eluted from HLB cartridges and evaporated to dryness under a nitrogen stream. Then, 50 μl of deionized water was added to the tube and redissolved.

### Ussing Chamber Experiments

The distal colon was cut longitudinally along the mesenteric border. The serosa, muscularis and submucosa layers were stripped away with fine forceps to prepare a mucosa sample. Stripped mucosa was mounted in a modified Ussing Chamber in a tissue holder (Easy Mount Chamber; Physiologic Instruments, San Diego, CA, United States) with an aperture surface area of 0.3 cm^2^. Each sample was bathed bilaterally in Krebs-Henseleit solution (KHS). A short-circuit current was measured *in vitro* in Ussing chambers. Transepithelial PD was then clamped at 0 mV, and the short-circuit current (*I*_*SC*_) was recorded with a VCCMC6 voltage-current clamp amplifier (Physiologic Instruments, San Diego, CA, United States). Transepithelial resistance (TR) (Ω^∗^cm^2^) was measured by altering the membrane potential in a stepwise fashion (0.1 mV) and calculated from the ohmic relationship.

### Solutions and Chemicals

Krebs-Henseleit solution (KHS) (mmol/L): NaCl, 117; KCl, 4.7; MgCl_2_, 1.2; KH_2_PO_4_, 1.2; NaHCO_3_, 24.8; CaCl_2_, 2.5; and glucose, 11.1. The solution was bubbled with 95% O_2_ and 5% CO_2_ to maintain a pH value of 7.4. Na^+^ free: NMDGCl 117; KCl 4.7; MgCl_2_.6H_2_O 1.2; KH_2_PO_3_ 1.2; choline HCO_3_ 25; CaCl_2_.2H_2_O 2.56; GS 11.1.

The indomethacin, tetrodotoxin (TTX), DPC, amiloride, and forskolin, were purchased from Sigma (St. Louis, MO, United States). Stock solutions of all the above chemicals were dissolved in DMSO. The final DMSO concentration never exceeded 0.1% (v/v). Preliminary experiments indicated that the vehicle did not alter any baseline electrophysiological parameters.

### Western Blotting

Tissue samples were collected from the colon mucosa of the control and high-lactose diets. Tissues were homogenized and sonicated in cold lysis buffer with protein inhibitiors and phosphatase inhibitors (Beyotime Biotechnology, Beijing). After 30 min standing in 4°C, the samples were then centrifuged at 12,000 × *g* for 30 min at 4°C. The pellets were discarded, and the supernatant was used for blotting. The proteins (50 μg) were separated via 8 or 10% SDS/PAGE. The blot was washed with Tris-buffered saline containing Tween-20 (TBST) and incubated overnight at 4°C with polyclonal primary antibodies against MCT1, sMCT1, NKCC1, and CFTR (Supplementary Table S1) and the rabbit polyclonal anti-actin antibody (Supplementary Table S1). After washing with TBST, the membranes were incubated with secondary antibodies (Supplementary Table S1).

### Immunostaining

The specimens were fixed with 4% paraformaldehyde for 10 min at 25°C, rinsed three times, and incubated with blocking serum for 1 h and anti-MCT, anti-sMCT1, anti-NHE3, anti-NKCC1 and anti-CFTR antibodies overnight at 4°C. After washing with PBS, the specimens were incubated with secondary antibodies. All antibodies in the experiments were shown in the Supplementary Material (Supplementary Table S1). Images were obtained using a fluorescence microscope (Leica DM2500, Germany) with 20× or 40× dry lens.

### Statistical Analyses

All data are presented as the mean ± SEM. Differences between three groups were evaluated using ANOVA with Prism software (version 9.1, SAS Institute, Inc.), and those between two groups were assessed using the *t*-test. The Wilcoxon *t*-test was used to compare microbial communities between two groups. Principal component analysis (PCA) plots of bacterial populations were created using Metaboanalyst 3.0. LEfSe analysis of the treatment group was performed on the basis of the results of the Wilcoxon tests, and the threshold on the logarithmic linear discriminant analysis (LDA) score was 3. Significance was defined as *P* < 0.05.

## Results

### Effect of IHLD-Induced Diarrhea on the Fecal Form, Fecal Fluid Content and Weight

After 30% lactose administration for 48 h, we observed that the majority of rats began to produce wet, loose and yellowish stools. On the 7th day and 14th day, we changed to 40 and 50% lactose and found profuse, watery stools visibly containing lots of mucus with or without a structural pellet form (Table 1). Compared to 30% lactose for 3 weeks, the graded increase in the concentration of lactose avoided the adaptation of rats to lactose and exhibited considerably more diarrhea than the same concentration of 30% lactose for 3 weeks. From our data, IHLD-fed rats could induce persistent and chronic diarrhea compared with only 30 or 40% lactose for 3 weeks, which can avoid adaptation to lactose in rats (Table 1). In the first week, the majority of diarrheic rats (80 and 90%) showed severe diarrhea with profuse, watery stools containing mucus after 30 and 40% lactose diet. In the 3rd week, the fecal characteristics of most rats in the 30 or 40% lactose group showed loose and soft stool pellets, suggesting that diarrhea began to improve. 20% of rats in the IHLD group produced profuse, loose, wet and mucoid stools, and 40% of rats in the IHLD group produced many loose, yellowish, wet stools with or without the structural form of pellets.

**TABLE 1:** Comparison of the severity of diarrhea after single high lactose (30 or 40%) or incremental high lactose diet (30%/40%/50%) feeding for 3 weeks.

Diets	Form of stool after 48 h to 21st day				
	Watery diarrhea + mucous (incontinence or profuse)	Loose stools + mucous (non-structural stool)	Loose stool with structural form of pellet	Soft formed pellets	Hardened normal pellets
Standard chow diet	–	–	–	++	++++
30% lactose diet 1st w	+++	++	+	+	–
40% lactose diet 1st w	++++	++++	–	–	–
IHLD 1st w	+++	++	+	+	–
30% lactose diet 2nd w	–	+	+	+	–
40% lactose diet 2nd w	++	++	++	–	–
IHLD 2rd w	+	++	+	+	–
30% lactose diet 3rd w	+	–	++	++	+
40% lactose diet	–	+	++	+	–
3rd w IHLD 3rd w	+	+	++	+	–

*IHLD: incremental high-lactose diet 1st w: the first week, 2nd w: second week, 3rd w: third week. Qualitative assessment score or classification: – condition not observed, + is the observed once/day; ++ is the observed twice/day. +++ is the observed 3–4 times/day, ++++ is the abundantly observed and +++++ is the almost exclusively observed.*

**FIGURE 2:**
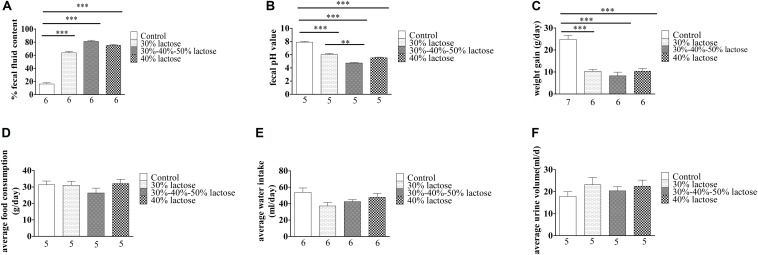
Summary data on the characteristics of animals after 30 and 40% lactose and IHLD (30%/40%/50%) lactose diet feeding for 21 days. **(A)** The three kinds of HLD models caused dramatic increases of 3-fold, 4.2-fold, and 4-fold of the fecal fluid content, respectively. **(B)** Fecal pH significantly decreased after three kinds of HLD feeding. **(C)** Three kinds of HLD models caused significant weight loss compared with the standard chow diet (control, *P* < 0.001). **(D)** IHLD (30%/40%/50%) reduced the average food consumption, but 30 and 40% lactose diet food consumption did not significantly alter it. **(E)** Water intake was decreased after IHLD, but there were no significant differences between IHLD and control. **(F)** IHLD did not affect average urine compared with control rats. The numbers under the bar graph indicate the rat number. **P < 0.01, ***P < 0.001.

The IHLD caused a significant increase in fecal fluid content, as fluid content constituted 79% of stool mass compared with 16% in pellets produced by rats fed the standard diet (Figure 2A; *P* < 0.001). Furthermore, IHLD significantly decreased the fecal pH value compared with that of control rats (Figure 2B, *P* < 0.001). In addition, an IHLD significantly decreased weight (Figure 2C), similar to previous studies (Liuzzi and Hevia, 1998; Boakye et al., 2012). The everyday average food consumption and water intake were mildly decreased compared to those of the control group (Figures 2D,E). The average urine volume were almost same among the four groups (Figure 2F).

### Microbial Dysbiosis After Incremental High-Lactose Diet

**FIGURE 3:**
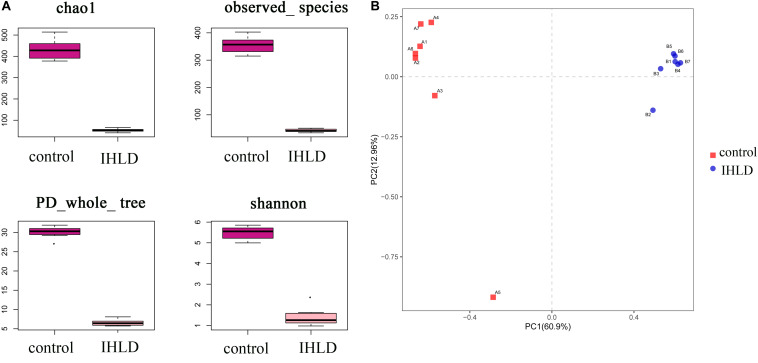
The diversity, richness, and microbial composition in the IHLD group differ from those of the control. **(A)** OTU richness and diversity indices. **(B)** Principal component analysis of cecal microbiota on the OTU level.

Incremental high-lactose diet induced significant alterations in the composition of the luminal microbiota of the cecum by 16S rRNA-Amplicon sequencing. A total of 14 samples from the two groups were analyzed by high-throughput pyrosequencing, which generated 1,133,201 clean tags. After IHLD, the Shannon and Chao 1 indexes revealed that the diversity and richness of the microbial communities were significantly lower than those in the control group (Figure 3A). Briefly, the results of PCA (Principal component analysis) showed that the composition of the luminal microbiota of IHLD formed a totally different cluster in the ordination plot, suggesting that IHLD caused major changes in the gut microbiome profiles (Figure 3B).

**FIGURE 4:**
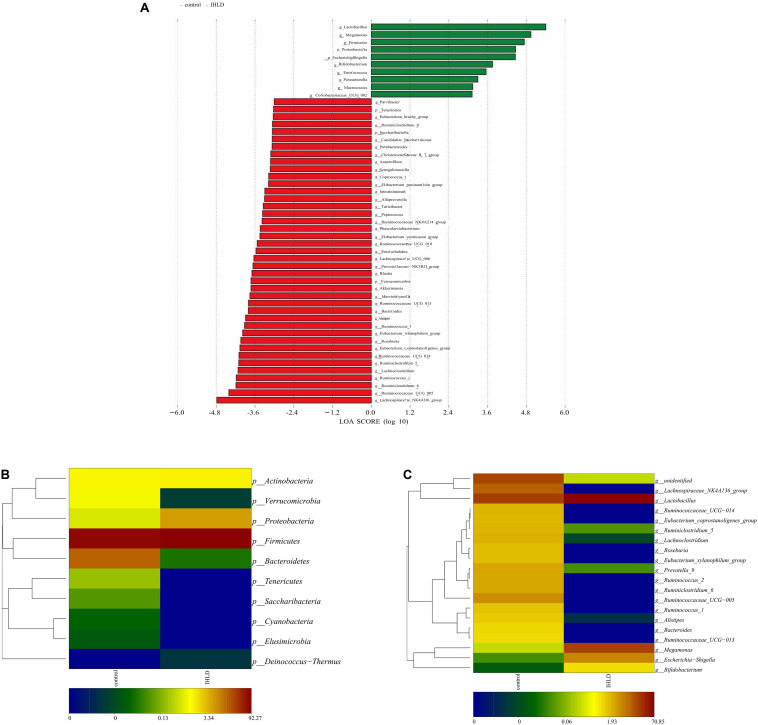
Changes in the composition of luminal microbiota at the phylum and genus levels between the control and IHLD groups. LEfSe analysis of the differences between two groups at the phylum and genus levels. **(A)** Heatmap of the relative abundances of predominant phyla **(B)** and genera [top 20, **(C)**]. The heatmap plot depicts the relative percentage of each bacteria depicted by color intensity with the legend indicated at the bottom of the figure.

Univariate analysis (LDA effect size) was performed to identify the key phyla and genera that exhibited large alterations in microbial composition after IHLD (Figure 4A). At the phylum level, the abundance of Bacteroidetes (16.54% versus 0.029%) and Verrucomicrobia (1.01% versus 0.0036%) significantly decreased, whereas that of Proteobacteria significantly increased after IHLD (0.58% versus 6.28%) (Figure 4B and Supplementary Table S2). At the genus level, the abundance of 139 genera showed significant differences between the two groups. The IHLD group showed a marked increase compared to the control group in the relative abundances of Bifidobacterium (0.004% versus 1.12%), Escherichia-Shigella (0.02% versus 5.7%), Megamonas (0.23% versus 20.33%), and Lactobacillus (21.85% versus 70.85%) (Figure 4B and Supplementary Table S2). In contrast, the concentrations of 59 genera were decreased to zero in the IHLD group, as shown in the Supplementary Table S2, including SCFA-producing bacteria such as Ruminococcaceae UCG-013 (Figure 4C), Lachnospiraceae UCG-001 and Lachnospiraceae FCS020 group, also including lactose-fermenting Roseburia species increased by administration of a highly purified short-chain galactooligosaccharide (GOS) (Azcarate-Peril et al., 2017).

**FIGURE 5:**
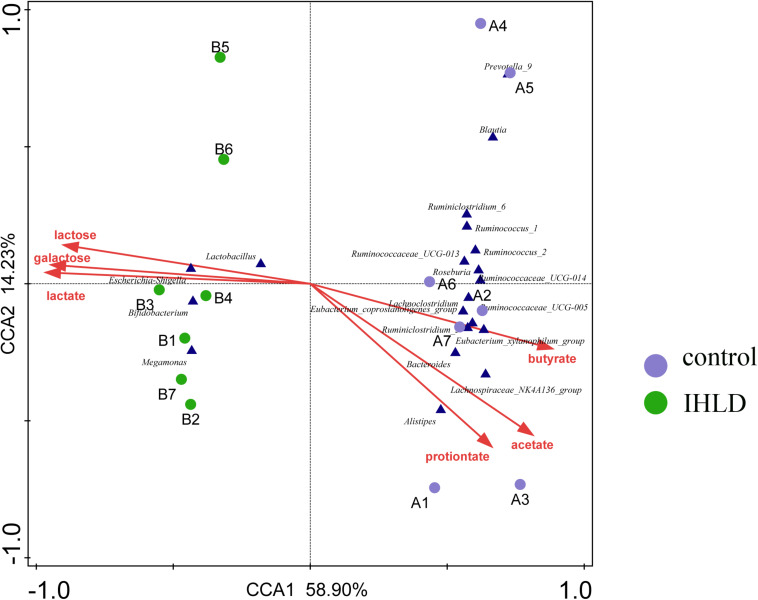
Constrained correspondence analysis revealing the correlations between the relative abundance of the microbial genus and the concentrations of lactose and lactose fermentation products.

Correlation analyses were performed to identify the relationships between microbial genera and lactose fermentation products including lactose, galactose, lactate and SCFAs. The results showed the relative abundances of four genera, including Lactobacillus, Bifidobacterium, Megamonas and Escherichia Shigella, showing a strong relationship with lactose, galactose and lactate (Figure 5). Among them, the relative abundance of lactobacilli, as the best-known lactic acid-producing bacteria, was 3.3-fold higher in IHLD than in the control. Another 16 genera, especially Allobaculum, Akkermansia, Lactobacillus, Bifidobacterium, and Bacteroides, with very low relative abundances showed a close association with the concentration of SCFAs.

### SCFA, Lactose, Galactose and Lactic Acid Quantification in the Cecal Luminal Contents

**FIGURE 6:**
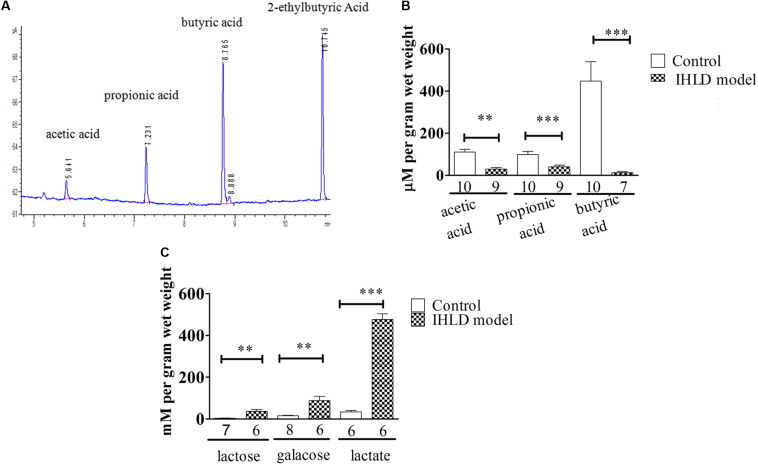
Total amounts of short-chain fatty acids (SCFAs) and lactose, galactose and lactic acid in the cecum luminal contents. **(A)** Chromatogram of SCFAs, including acetate, propionate, butyrate and 2-ethylbutyric acid (internal standard). **(B)** SCFAs in the cecum were significantly decreased in IHLD compared to control (**P < 0.01, ***P < 0.001). **(C)** The concentrations of lactose, galactose and lactic acid were significantly increased in the IHLD group compared to the control group. The numbers under the bar graph indicate the rat number.

The SCFA concentration is high in the cecum and proximal colon since SCFAs are produced by microbial fermentation in the cecum (Tan et al., 2014; Kuwahara et al., 2018). Therefore, we investigated the concentration of SCFAs in the cecum. Lactose, galactose, lactic acid and SCFAs were quantified in cecal contents in the control and model groups. Interestingly, a significant decrease in the concentration of SCFAs was observed in the intestinal contents in the IHLD group in contrast to the control group. Acetate, propionate and butyrate were 140% lower in the cecum in the model group than in the control group, as presented in Figures 6A,B, suggesting that SCFA production was lower in the colon. In contrast, lactose, galactose and lactic acid concentrations were 10-, 6-, and 15-fold higher in the IHLD group, respectively than in the control group (Figure 6C). The results suggested that the high concentrations of lactose, lactic acid and galactose in the colon lumen but not SCFAs led to hyperosmosis in the colon, which would be the major reason for diarrhea.

### Expression and Location of MCT and sMCT1 in the Colon of the IHLD Group and Control Group

**FIGURE 7:**
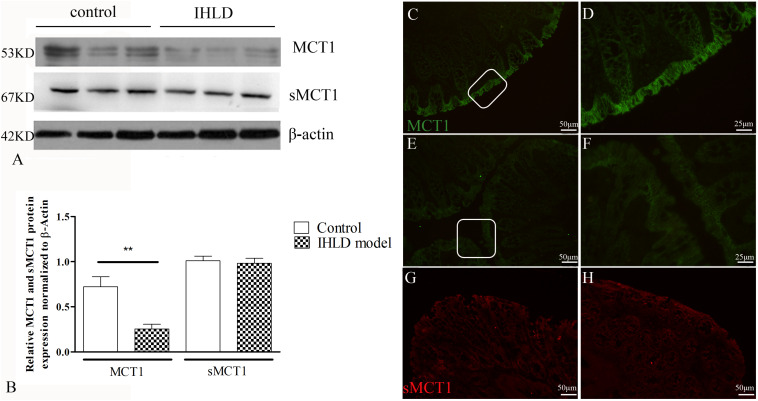
MCT1 and sMCT1 expression and location after IHLD. Western blot analysis of total MCT1 and sMCT1 in the colon of the control and IHLD models. Images are representative immunoblots of MCT1, sMCT1, and β-actin from five animals in each group **(A)**; densitometric analysis of MCT1 and sMCT1 abundance in colon between control and IHLD model group (**P < 0.01) **(B)**. Immunofluorescence staining of MCT1 and sMCT1 in colonic sections of the control **(C,D,G)** and IHLD model **(E,F,H)**. Panels **(D,F)** are higher magnification images of the white rectangles in panels **(C,E)**, respectively. The immunoblots are representative of the results obtained from five animals in the model group. Scale bar = 50 μm.

MCT1 is an H^+^-coupled transporter expressed in the apical membrane and basolateral membrane of the colonic epithelium (Ritzhaupt et al., 1998; Kirat et al., 2009), mediating SCFA influx from the lumen and SCFA efflux into the blood (Iwanaga et al., 2006). The western blot results showed that the expression level of MCT1 protein in the colon of the IHLD group was lower by 65% than that in the control group (Figures 7A,B). Moreover, the number and intensity of MCT1 immunopositive cells were greatly decreased in lactose-fed rats (Figures 7E,F) compared with control animals (Figures 7C,D), confirming the western blot results.

sMCT1 is a Na^+^-coupled transporter expressed solely on the apical membrane of the colonic epithelium that mediates SCFA influx from the lumen (Ritzhaupt et al., 1998; Kuwahara et al., 2018). The western blot results showed that the expression level of sMCT1 did not change in IHLD rats compared to the control rats, as shown in Figure 7A, consistent with the immunofluorescence results (Figures 7G,H).

### Involvement of Na^+^ Absorption and Anion Secretion in the Colon of the IHLD-Induced Diarrhea Group

**FIGURE 8:**
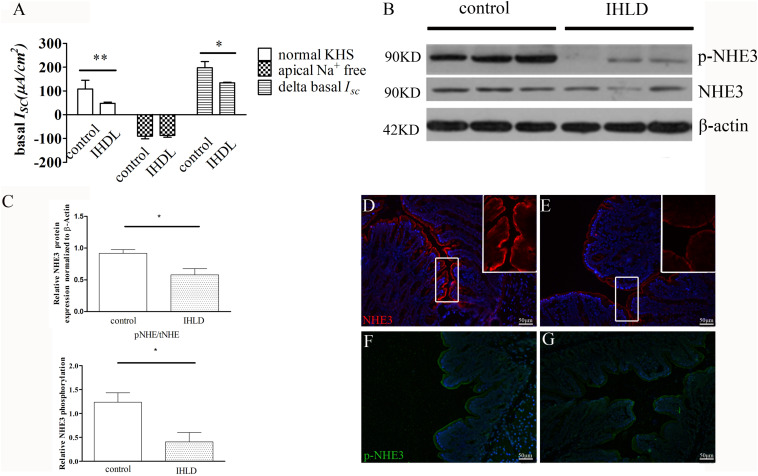
Decreased NHE3 expression and phosphorylation levels and Na^+^ absorption involved in lactose-induced chronic diarrhea. Baseline *I*_*SC*_ current and delta basal *I*_*SC*_ in colonic epithelium between the control and IHLD groups (*P < 0.05, **P < 0.01) **(A)**. Western blot analysis of total NHE3 and phosphorylated NHE3 in the colon of the control and IHLD models. **(B,C)** Images are representative immunoblots of NHE3 and phosphorylated NHE3 from five animals in each group **(B)**. densitometric analysis of NHE3 and relative NHE3 phosphorylation (pNHE3/NHE3) in colon between control and IHLD model group (*P < 0.05) **(C)** Immunofluorescence staining of NHE3 and phosphorylated NHE3 in the colon between the control **(D,F)** and model **(E,G)** groups. The right inset is zoomed image of white rectangle **(D,E)**. Scale bar = 50 μm.

In order to investigate the basal and stimulated ion transport in the colonic epithelium of the IHLD group, a Ussing Chamber experiment was performed. In short circuit current recording, the basal current represents a summation of all ionic currents across the epithelium (Xue et al., 2007; Clarke, 2009). The basal *I*_*SC*_ was lower in the IHLD-model group (IHLD: 48 ± 5.15 μA/cm^2^ versus control: 108 ± 37.23 μA/cm^2^, *n* = 10, Figure 7A). In apical Na^+^-free KHS, the basal *I*_*SC*_ without apical Na^+^ absorption in the colonic mucosa of the control group was similar to that of the IHLD group (IHDL: −86.42 ± 7.8 μA/cm^2^ versus control: −89.69 ± 11.41 μA/cm^2^, *n* = 11). However, the Δbasal *I*_*SC*_ (subtract the basal *I*_*SC*_ in apical Na^+^-free KHS from the basal *I*_*SC*_ in normal KHS) in the colonic mucosa of the IHLD group was significantly decreased (IHLD 134.4 ± 2.65 μA/cm^2^ versus control: 197.8 ± 25.82 μA/cm^2^, *n* = 10, Figure 7A), suggesting that Na^+^ absorption was lower in the IHLD group. After apical treatment with the ENaC inhibitor amiloride, a similar inhibition of basal *I*_*SC*_ was observed in the control and IHLD groups, excluding the ENaC involved in the ion transport of the IHLD group (IHLD: −6.54 ± 1.73 μA/cm^2^ versus control: −6.98 ± 2.22 μA/cm^2^, Supplementary Figure S1). As Figure 8 shown, the results of western blotting and immunostaining showed that the level of total protein expression and phosphorylation of NHE3 protein-mediated Na^+^ absorption in the colon were downregulated by 37 and 67% respectively, in the model group compared with the control group (Figures 8B–G), which was responsible for the low Na^+^ absorption.

**FIGURE 9:**
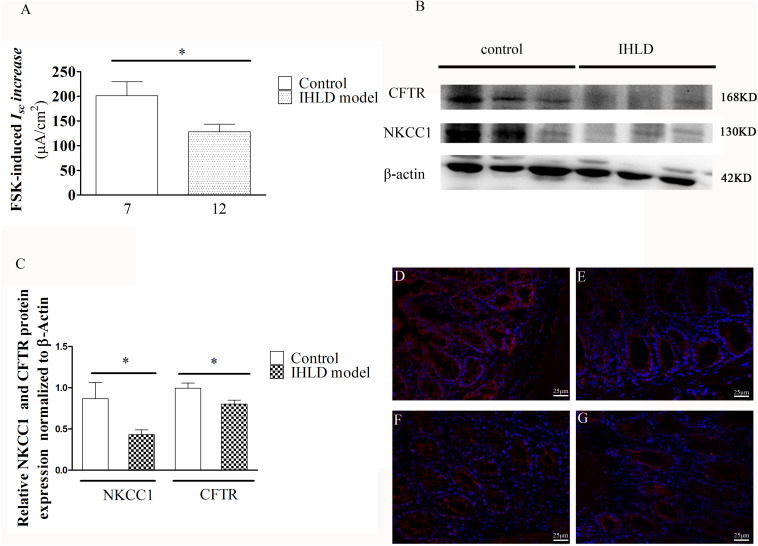
Mucosal secretory responses and expression of CFTR and NKCC1 in rats after IHLD/SD feeding for 21 days. Forskolin-stimulated *I*_*SC*_ **(A)** in the colonic epithelium (*P < 0.05). The numbers under the bar graph are the samples number from four different rats. Western blot analysis of total NKCC1 and CFTR in the colon of the control and IHLD models. (*P < 0.05). **(B,C)** Images are representative immunoblots of CFTR and NKCC1 from five animals in each group. Immunofluorescence staining of NKCC1 **(D,E)** and CFTR **(F,G)** in the colon between the control **(D,F)** and model **(E,G)** groups. Scale bar = 50 μm.

Furthermore, the cAMP elevator forskolin (1 μM, basolateral)-induced *I*_*SC*_ increase in the colon of the IHLD group was lower than that in the control group (IHLD: 128.3 ± 15.3 μA/cm^2^ versus control: 201.4 ± 28.31 μA/cm^2^, Figure 9A). The inhibition of basal *I*_*SC*_ induced by apical treatment with a known CFTR inhibitor DPC (diphenylamine-2, 2′-dicarboxylic acid), was significantly higher in the control group than in the IHLD group, suggesting that CFTR-mediated Cl^–^ secretion under basal conditions was decreased in the IHLD group compared with the control group (IHLD: −1.12 ± 1.74 μA/cm^2^ versus control: −8.10 ± 2.14 μA/cm^2^, Supplementary Figure S1). The western blot and immunostaining results showed that the protein expression levels of NKCC1 and CFTR in the colonic mucosa were decrease by 50 and 24%,respectively, than those in the control group, contributing to the lower basal and stimulated ion secretion in the model group (Figures 9B–G).

### Discussion

Lactose intolerance (LI) appears to be mainly associated with unabsorbed lactose in the small intestine passing into the colon and being metabolized by colonic bacteria to organic acids, including lactic acid and short-chain fatty acids. The organic acids escaping absorption by the colon remain in the colonic lumen and lead to osmotic diarrhea. Previous studies have shown that lactose-induced diarrhea results, in large part, from the combination of net fluid secretion by the stomach and small intestine and interference with net fluid absorption in the colon (Christopher and Bayless, 1971; Hammer et al., 1989).

In our study, we established a chronic diarrhea model using lactose with a gradual increase in concentration for 3 weeks to investigate the mechanism of high lactose-induced diarrhea on organic acid production and ion transport of the colonic epithelium and NHE3, CFTR and NKCC1 expression combined with epithelial locations, which are associated with Na^+^ absorption and Cl^–^ secretion.

The concentrations of lactose, galactose and lactic acid in the cecum in the IHLD model group were higher than those in the control group, but the concentration of SCFAs was lower, suggesting that the major reason for hyperosmosis is due to high concentrations of lactose, galactose and lactic acid in the intestinal lumen, which could cause osmotic diarrhea. Furthermore, the concentration of lactic acid in the cecum content was much higher than that in the control group, which was responsible for the low pH in the feces. Adult rats, as mammals, have intestinal lactase activity that decreases quickly upon weaning, and research has shown that although residual lactase activity is present in adult rats, a high-lactose diet is poorly digested by adult rats (Alexandre et al., 2013; van de Heijning et al., 2015). The increase in galactose in the cecum contents after chronic feeding with high lactose might be due to microbiota fermentation. As expected, our results showed that significant differences were observed in the composition of cecum microbiota via high-throughput MiSeq sequencing after IHLD. A previous study (Francavilla et al., 2012) investigated the influence of lactose on the composition of the gut microbiota and the SCFAs of infants with cow’s milk allergy. Similar to our findings, lactose intake can induce an increase in Bifidobacteria and Lactobacilli and a decrease in the numbers of Bacteroides, Prevotella and Clostridia. In addition, we also found that the increased abundances of another two genera, Escherichia-shigella and Megamonas, were positively related to the concentrations of lactose, galactose and lactic acid and might be responsible for the IHLD-induced diarrhea. They also measured SCFA levels and found that the concentration of SCFA increased after addition of lactose, which was the opposite result of our study because of the differences between from infants and adults. In addition, we found the loss of important SCFA-producing microbes, such as lachnospiraceae NK4A136 group and ruminococcaceae UCG-005, after IHLD, which might lead to the lower concentration of luminal SCFAs, consistent with a recent study on a high-sugar diet (Laffin et al., 2019). Another recent study (Kamphuis et al., 2019) showed that oral gavage with lactose at a modest dose of 5 mg/day for 3 weeks did not induce alterations in the fecal microbiota of mice, which was different from our study because of the low dose of lactose without significant malabsorption.

In our results, we found that the concentrations of SCFAs in the cecal contents of the model group was significantly decreased compared with that of the control group, which was different from a previous study that showed that an increase in SCFAs resulted in acute ingestion of lactose *in vivo* and *in vitro* (He et al., 2006; Windey et al., 2015). Few studies have focused on the effect of chronic lactose intervention on SCFAs, and a recent study (Zhai et al., 2018) found no significant difference in the SCFA concentration of feces after 3 weeks of lactulose intervention in mice, suggesting a different mechanism between acute and chronic ingestion. In the colon, SCFAs are absorbed by MCT1 and sMCT1 in the colon epithelium. Our results showed that an unaltered expression level and differential distribution of sMCT1 were observed between the control group and IHLD group. However, MCT1 expression was significantly decreased, showing that its low expression level might be caused by lower SCFAs in the lumen, which is also reported to be regulated by butyrate (Borthakur et al., 2008, 2012).

Currently, there is growing evidence indicating that SCFAs are a product of bacterial fermentation involved in the regulation of ion transport (Yajima et al., 2011; Tan et al., 2014; Kuwahara et al., 2018). Previous studies demonstrated that luminal addition of propionate stimulates chloride secretion, which function as a lubricant for the movement of luminal contents in the colon (Yajima et al., 2011). It has been reported that NHE3 mediates Na^+^ absorption in the colon, and our data showed that its expression and phosphorylation level were significantly decreased in the model group, confirming a decrease in Na^+^ absorption. The results of the Ussing Chamber demonstrated that when apical Na^+^ was removed, the delta basal *I*_*SC*_ in the IHLD group was significantly decreased, suggesting that Na^+^ absorption was involved in the basal current. It is generally accepted that the absorption of Na^+^ and Cl^–^ in the colon is mutually interdependent, electrically neutral by largely apical Na/H exchange and Cl/HCO_3_ exchange processes, primarily involving the Na/H exchanger NHE3 and the Cl/HCO_3_ exchanger (Walker et al., 2008). The evidence from the present study showed that Na^+^ absorption and NHE3 protein expression and phosphorylation were decreased in IHLD rats compared with control rats, but the RNA level of NHE3 and the change in the expression of the Cl/HCO_3_ exchanger involving Cl^–^ absorption still need to be investigated. To the best of our knowledge, no previous report has found that NHE3 is involved in lactose-induced diarrhea and might be a novel target for the attenuation of lactose-induced diarrhea.

In this study, we used the chronic diarrhea model induced by giving high lactose for 3 weeks, which could employ a compensatory mechanism to adapt to the diarrhea situation. A previous study showed that rats adapted to a lactose diet and that the size of the cecum returned to normal, but its mechanisms are not clear (Lawrence et al., 1956; Szilagyi, 2015). Our results showed that basal current and FSK-induced Cl^–^ secretion in the model group were lower than those in the control group, which can result in lower luminal anionic secretion and associated luminal transport of water. Furthermore, the NKCC1 and CFTR expression levels in the colon mucosa in the model group decreased compared with those in the control group, resulting in a decrease in basal and FSK-induced *I*_*SC*_. We considered it to have resulted from the compensatory mechanism. In the duodenum, luminal SCFAs induced HCO${}_{3}^{-}$ secretion (Akiba et al., 2015). Our study showed that lower luminal SCFAs decrease Cl^–^ secretion, but the mechanism should be investigated further.

**FIGURE 10:**
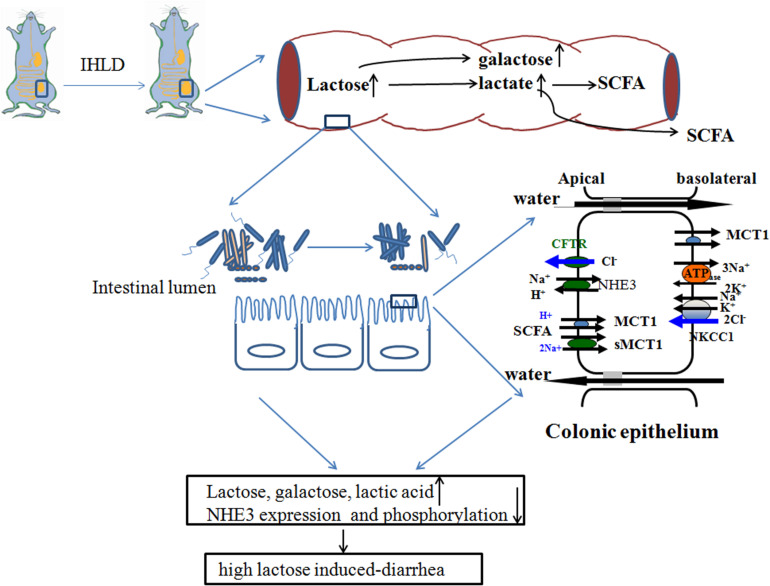
Schematic diagram of lactose-induced chronic diarrhea. Amounts of lactose, galactose and lactic acid remaining in the cecum resulted in microbiota dysbiosis, which was related to low lumen SCFAs. Low NHE3 expression and phosphorylation levels in the colonic mucosa of the IHLD group contributed to a decrease in Na^+^ absorption and accumulation in the lumen, and a decrease in stimulated Cl^–^ secretion would compensate for water and electrolyte loss during the diarrhea process.

Taken together, IHLD feeding to rats caused a persistent state of diarrhea, and the cecum tripled in size. The concentrations of lactose, galactose and lactic acid were markedly increased in the cecal luminal contents, contributing to lactose intolerance-induced high osmosis diarrhea. Meanwhile, the lumen SCFA concentration of the cecal contents was significantly decreased. This was associated with depletion of the Lachnospiraceae NK4A136 group and Ruminococcaceae UCG-005 and an increase in the relative abundance of Lactobacillus, escherichia-shigella and megamonas in the cecal microbiota. In addition, the microbiota diversity in the colon was found to be lower in the cecum of IHLD rats compared with control rats. Concomitantly, the expression of monocarboxylate transporter 1 was decreased in the colonic mucosa of the IHLD group. Low NHE3 expression and phosphorylation levels in the colonic mucosa of the IHLD group contributed to the Na^+^ absorption and accumulation decrease in the lumen, and the stimulated Cl^–^ secretion decrease would be a compensatory effect for water and electrolyte loss during the diarrhea process (Figure 10). The present study provides new evidence for the physiopathological mechanism of lactose intolerance-induced high osmosis diarrhea and new ideas for therapy through modulating the gut microbita and Na^+^ absorption.

## Data Availability Statement

The original contributions presented in the study are publicly available. This data can be found here: https://www.ncbi.nlm.nih.gov/bioproject/PRJNA611011.

## Ethics Statement

The animal study was reviewed and approved by the Animal Care Ethics Committee of Xiyuan Hospital (Permission code: 2019XLC004-2).

## Author Contributions

HX, FW, and XT conceived the study. HX designed the experiment, wrote the manuscript, and performed the western blotting and immunostaining. MZ and JM made the animal models and performed the Ussing Chamber experiments. HX quantified SCFAs. HX and TC analyzed the data. FW and XT supervised the research. All authors contributed to the article and approved the submitted version.

## Conflict of Interest

The authors declare that the research was conducted in the absence of any commercial or financial relationships that could be construed as a potential conflict of interest.

## Supplementary Material

FIGURE S1 Comparison of effect of DPC or amiloride on the basal *I*_*SC*_ current between control and IHLD group. Values are means ± SEM; *P* < 0.05.

TABLE S1 Antibodies for western and immunofluorescence.

TABLE S2 Information of statistical test analysis of phylum and genus level between control and IHLD group.
