# Supplementary material for: Lactose-Induced Chronic Diarrhea Results From Abnormal Luminal Microbial Fermentation and Disorder of Ion Transport in the Colon
Source: Front Physiol. 2020 Jul 29;11:877. doi: 10.3389/fphys.2020.00877 (PMC7403511; doi:10.3389/fphys.2020.00877)
Supplement: TABLE S2 — Information of statistical test analysis of phylum and genus level between control and IHLD group. [file Data_Sheet_1.PDF]

Antibodies for western and immunofluorescence

Primary Antibody	Dilution	Host species	Source/Catalog no.	Secondary antibody	Dilution	Source/Catalog no.
MCT1	1:1000 (WB)	chicken	Chemicon/ AB1286-I	HRP-conjugated goat anti-chicken Ig Y	1:5000	Immunoway/RS2101
	1:2000(IF)			Alexa Fluor 488 goat anti-chicken IgY	1:200	Immunoway/RS3201
sMCT1	1:1000(WB)	rabbit	Proteintech/21433-1-AP	Peroxidase-conjugated goat anti-rabbit Ig G	1:5000	Proteintech/SA00001-2
	1:50(IF)			Alexa Fluor 594 goat-anti rabbit IgG	1:500	Abcam/ab150080
NHE3	1:100(WB)	rabbit	Alomone labs/ANX-033	Peroxidase-conjugated goat anti-rabbit Ig G	1:5000	Proteintech/SA00001-2
	1:200(IF)	rabbit	Alpha Diagnostic Intl/ NHE31-A	Alexa Fluor 594 goat-anti rabbit Ig G	1:5000	Abcam/ab150080
p-NHE3	1:1000(WB)	mouse	Invitrogen/MA1-46415	Peroxidase-conjugated goat anti mouse IgG	1:5000	Proteintech/SA00001-1
	1:20(IF)	mouse	Invitrogen/MA1-46415	Alexa Fluor 488 goat ani mouse IgG	1:1000	Abcam/ab150113
CFTR	1:200(WB)	rabbit	Alomone labs/ACL-006	Peroxidase-conjugated goat anti-rabbit Ig G	1:5000	Proteintech/SA00001-2
	1:50(IF)			Alexa Fluor 594 goat-anti rabbit IgG	1:500	Abcam/ab150080
NKCC1	1:200(WB)	rabbit	Alomone labs/ACL-006	Peroxidase-conjugated goat anti-rabbit Ig G	1:5000	Proteintech/SA00001-2
	1:50(IF)			Alexa Fluor 594 goat-anti rabbit IgG	1:500	Abcam/ab150080
β-actin	1:5000(WB)	mouse	Proteintech/66009-1	Peroxidase-conjugated goat anti mouse IgG	1:5000	Proteintech/SA00001-1
